# Construction of a microbial abundance prognostic scoring model based on intratumoral microbial data for predicting the prognosis of lung squamous cell carcinoma

**DOI:** 10.1515/biol-2025-1169

**Published:** 2025-10-23

**Authors:** Rongxin Shang, Chao Yuan, Xiaohua Liang, Yuhui Yun, Jianbo Jia, Jiakuan Chen, Guoliang Han

**Affiliations:** Department of Thoracic Surgery, Tangdu Hospital, The Air Force Military Medical University, Xi’an, Shaanxi, 710038, PR China

**Keywords:** intratumoral microbiota, lung squamous cell carcinoma, prognosis

## Abstract

The development of lung squamous cell carcinoma (LUSC) is associated with the intratumoral microbiota. To facilitate faster clinical decisions and predict patient prognosis, we constructed an intratumoral microbial abundance prognostic scoring (MAPS) model for LUSC and analyzed its prognostic performance. Data on the LUSC tumor microbiome, patient survival, and clinical information were downloaded from The Cancer Microbiome Atlas and The Cancer Genome Atlas databases. Differentially abundant microbial genera in LUSC tumors were analyzed, and their prognostic value was evaluated. The differential abundance of key genera in the MAPS model was validated using lung adenocarcinoma (LUAD) tumors and normal tissues. Of 52 microbial genera with increased abundance and 437 with decreased abundance in LUSC tumors, 462 were highly related to the disease. Seven of 13 genera that were significantly related to prognosis were selected to construct the MAPS model. The MAPS risk grouping was identified as a prognostic risk factor for LUSC. Among the seven genera in the MAPS model, *Indibacter*, *Oceanospirillum*, *Thalassomonas*, and *Thermopetrobacter* differed in abundance between LUAD tumors and normal tissues and may be the key intratumoral microorganisms involved in LUSC and LUAD development. In conclusion, our MAPS model may be a powerful prognostic biomarker for LUSC.

## 1 Introduction

Cancer poses a heavy burden on the health, longevity, and quality of life of patients and their families. The risk factors for cancer include family genetics, lifestyle factors, obesity, smoking, nutritional deficiency, drinking, ultraviolet radiation, and sources of infection [1]. Microbial communities are commonly found in tumor tissues, and different tumor types have unique microbial communities [2]. Over the past few decades, many microorganisms have been extensively studied, and 11 types have been identified as grade 1 carcinogens [3]. The mucosal surfaces of the lungs, which have the largest surface area in the human body, are continuously exposed to various airborne microorganisms and environmental pollutants through inhalation. Most of the microorganisms that invade healthy human lungs belong to four phyla (Bacteroides, Firmicutes, Proteus, and Actinomycetes), with *Streptococcus*, *Neisseria*, *Hemophilus*, and *Fusobacterium* being the most abundant genera [4,5]. The characteristics of lung microbial communities vary according to the tissue and tumor type and depend on microbial community–host interactions [6,7]. Several studies have confirmed that many chronic respiratory diseases, such as asthma, chronic obstructive pulmonary disease, cystic fibrosis, and non-small cell lung cancer (NSCLC), are related to changes in microbial diversity in the respiratory tract [8,9].

Lung squamous cell carcinoma (LUSC) accounts for approximately 30% of all NSCLC cases [10]. Although smoking and environmental exposures are the most common risk factors for LUSC [11], the number of non-smoking patients with this NSCLC subtype has risen sharply over the past decade, suggesting the contribution of other additional but unidentified risk factors [12]. The diversity of intratumoral microorganisms is related to the clinical stage of the disease. Compared with lung adenocarcinoma (LUAD), LUSC with TP53 mutations has been found to be positively correlated with an abundance of *Acidovorax* [13].

Microbes are key regulators of carcinogenesis and the immune response to cancer cells, and cancer-causing immune cells have been linked to the composition of specific microbial groups [14]. The steady state of the lung immune system is not very stable because its interaction with the microbial community is dynamic and changes with age, heredity, and environmental exposure. Ecological imbalance in the lung microbial community leads to a disruption in lung homeostasis, rendering the host prone to lung diseases. At the same time, the abundance of the lung microbial community is significantly positively correlated with tumor growth [15]. The mechanisms underlying the emergence and progression of pulmonary microbial dysbiosis and the role that such microbiome imbalance plays in the development of lung diseases remain poorly understood. Next-generation sequencing technology provides a powerful tool for analyzing the diversity of tumor-associated microbial communities, enabling detailed characterization of the microbial composition in lung tumors and their alterations under specific conditions at the community or group level. Therefore, the aims of this study were to determine and establish the relationship between LUSC tumors and the intratumoral microorganisms, further explore the occurrence and development of LUSC, and establish new diagnostic and treatment methods for this malignant disease.

## 2 Materials and methods

### 2.1 Data source

LUSC-related patient survival data and clinical information were downloaded from The Cancer Genome Atlas (TCGA) database [16]. LUSC tumor microbiome data were downloaded from The Cancer Microbiome Atlas database [17]. To best characterize putative cancer-associated microbes, the potential effects of contamination must be reduced. Therefore, potential contaminants in this dataset were rigorously removed using *in silico* decontamination methods described in a previous study [17]. After Voom-SNM standardization, 1,553 genera were detected and quantified. The two groups of data were matched according to the sample number, and the tumor microbiome data of repeated samples were averaged. The LUSC dataset from TCGA included information on intratumoral microbiota abundance, age, sex, tumor stage, smoking, patient status, and overall survival (OS) for 82 tissue samples. Of these samples, 50 were tumor tissues and 32 were adjacent non-tumor tissues. Among the 50 tumor samples, 2 were from Black or African-American patients, 38 were from White patients, and 10 had no related race information. Of the 32 control samples, one sample was from Black or African-American patient, 21 samples were from White patients, and 10 samples had no related race information. The detailed information on these 82 samples is shown in Table S1.

### 2.2 Identification of differentially abundant genera

The differentially abundant genera between tumor and normal tissue samples were analyzed on the basis of the matched LUSC tumor microbiome data from TCGA. The threshold values for selecting differentially abundant genera were a *P*-value of less than 0.05 and |log2 fold change| of greater than 0.3 [18].

### 2.3 Screening LUSC-related modules

On the basis of the tumor microbiome data of the 82 samples in the LUSC dataset, the weighted gene co-expression network analysis (WGCNA; version 1.72.5) tool in the R package [19] was used to analyze 1,553 genera to identify highly coordinated microbiome modules. The pickSoftThreshold function in WGCNA was used for network topology analysis to calculate the soft-threshold power and scale-free fit index (ranging from 1 to 20). An appropriate soft-threshold value was selected on the basis of *R*² (scale-free fit index) being greater than 0.85. Next using the selected soft-threshold value, an adjacency matrix was computed and a topological overlap matrix was constructed. The values in this latter matrix reflect similarities in the co-regulatory relationships between the genera. The cluster tree was cut using a dynamic tree-cutting algorithm. The minModule Size parameter (minimum number of genera in each module) was set to aggregate the genera into modules. The eigengene for each module was calculated using principal component analysis. Finally, the eigengene module was used to compute the module-phenotype correlation. Modules with a *P*-value of less than 0.05 were selected as LUSC-related ones.

On the basis of the WGCNA results, the genera in the LUSC-related modules were selected to extract intratumoral microbiome abundance data from the disease samples. The correlation between genera was calculated using the Spearman algorithm, and the microbial co-regulatory network was constructed using Gephi (version 0.10.1) software [20].

### 2.4 Identification of key genera

A Venn analysis of the genera in the WGCNA module and differentially abundant genera was performed, and those that overlapped were considered key genera.

### 2.5 Construction and validation of a microbial abundance prognostic scoring (MAPS) model

On the basis of the clinical data (OS time and status) from tumor samples in the LUSC dataset of TCGA and corresponding intratumoral microbiome data, univariate Cox regression was applied to assess the prognostic significance of each genus using survival (version 3.5-8) in the R package. A *P*-value of less than 0.05 was set as the threshold to identify genera significantly associated with prognosis.

Stepwise multiple regression analysis for prognostic genera was used to calculate the Akaike information criterion (AIC). The feature genus was selected on the basis of the minimum AIC value, and a MAPS model was constructed as follows:

\[\text{MAPS}=\mathop{\sum }\limits_{i=1}^{7}(\text{abundance}\hspace{.5em}\text{of}\hspace{.5em}\text{microbe}\hspace{.5em}i)\hspace{.5em}\times \hspace{.5em}\text{coef}.]\]

All patient samples were grouped into high-risk (MAPS higher than the median value) and low-risk groups (MAPS lower than the median value) according to the median MAPS value. Kaplan–Meier and receiver operating characteristic (ROC) curves of 1-, 3-, and 5-year survival predictions were used to evaluate the performance of the MAPS model in predicting OS. Additionally, to comprehensively compare the performance of the MAPS model with other published signatures, we searched the PubMed database for published prognostic gene signatures associated with lung cancer. On the basis of the LUSC dataset of TCGA, a multivariate Cox regression model was constructed using the expression of signature genes and their corresponding coefficients, and the risk scores were subsequently calculated. The gene sets from studies where more than 10% of the genes were missing in the dataset were deleted. On the basis of the risk scores and patient survival information, the concordance index (C-index) of these signatures in predicting patient prognosis was analyzed using the CoxPH function. Finally, the C-indexes from these published signatures and the MAPS model were compared.

### 2.6 Prognostic value of the MAPS model

To explore the correlations between different clinical features and MAPS values, the rank-sum test was used to calculate the differences in different clinical features (age, sex, pathological_T, pathological_N, tumor stage, smoking, and patient status) and MAPS risk grouping between the tumor and normal samples. Univariate and multivariate Cox regression analyses were used to evaluate the prognostic value of MAPS risk grouping and clinical data (age, sex, pathological_T, pathological_N, tumor stage, and smoking), and the regression results were displayed in a forest plot drawn using forest plot (version 3.1.3). Factors with a *P*-value of less than 0.05 in both univariate and multivariate regression analyses were considered independent factors. On the basis of the independent factors, a nomogram was constructed using rms (version 6.8-0) in the R package to predict the survival rate of patients with LUSC [21]. Calibration curve and decision curve analyses were performed to evaluate the performance of the nomogram.

### 2.7 Abundance analysis of model genera

After downloading the LUAD tumor microbiome data from The Cancer Microbiome Atlas database, we extracted and analyzed the genus-level data of normal and tumor tissues from patients. The differences between tumor and normal tissues were calculated on the basis of the abundance data of the genera in the MAPS model, and the results were visualized using ggplot2 (version 3.5.0) in the R package.

### 2.8 Statistical analyses

The Spearman algorithm was used for correlation analysis and a correlation heat map was drawn. The box diagram was displayed using ggplot2 (version 3.5.0). The Wilcoxon rank-sum test was used to calculate the differences between groups. Hazard ratios and 95% confidence intervals were presented for all prognostic factors.

## 3 Results

### 3.1 Identification of differentially abundant microbial genera

**Figure 1:**
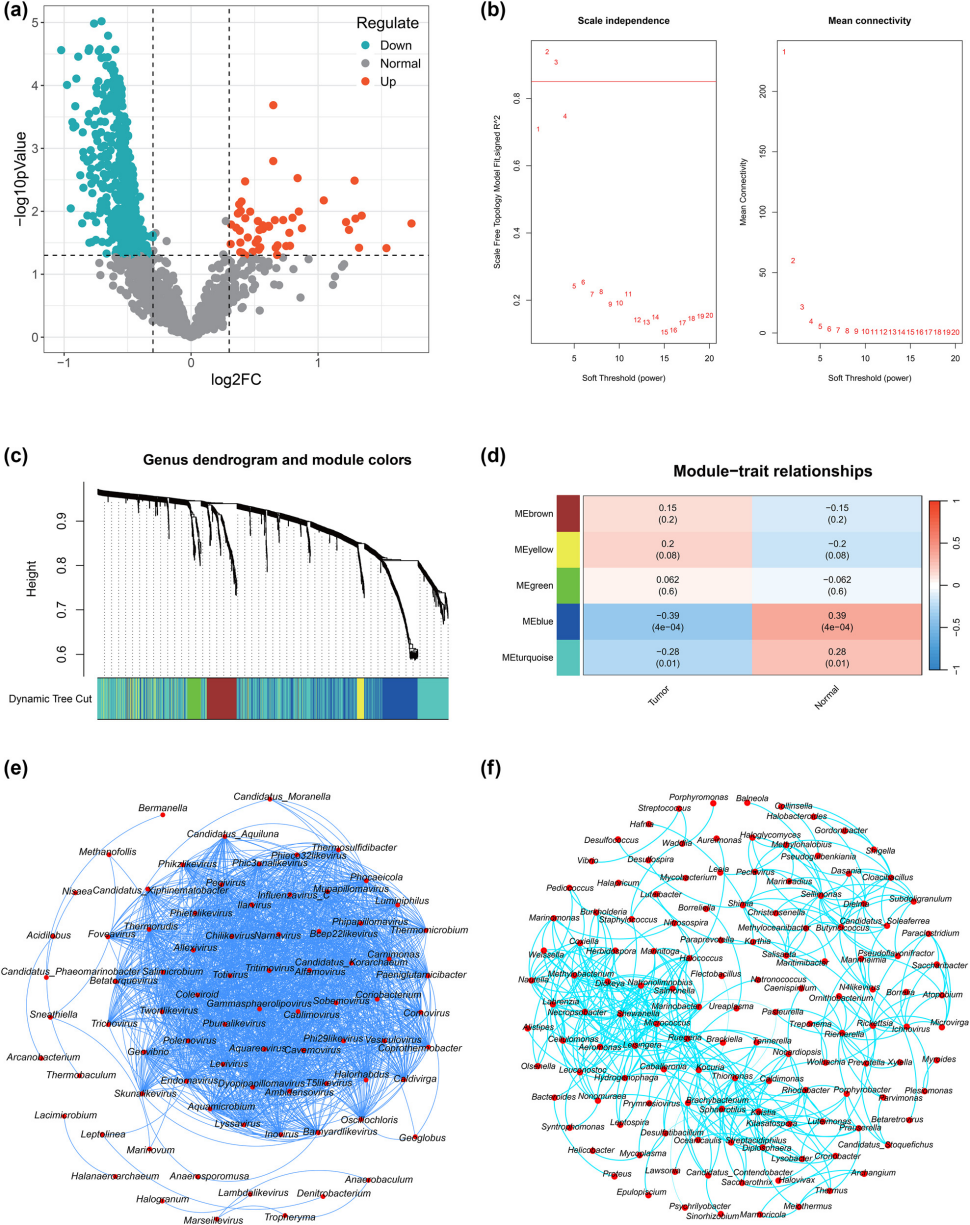
Identification of differentially abundant microbial genera. (a) Volcano map of differentially abundant microbial genera. (b) Scale-free fitting index. (c) Hierarchical clustering tree. (d) Correlation heat map between modules and traits. (e) Blue module belonging to the horizontal microbial interaction network. (f) Turquoise module belonging to the horizontal microbial interaction network.

The differential analysis of 1,553 genera revealed that 52 were increased and 437 were decreased in abundance in the tumor samples relative to their abundances in the control samples (Figure 1a).

### 3.2 Screening LUSC-related modules using WGCNA

The network topology analysis showed that the scale-free fitting index *R* ^2^ was 0.94 (Figure 1b). A topological overlap matrix was constructed according to the selected threshold, and the genera were divided into five modules. We analyzed the correlation between these modules and phenotypes. Among these, the turquoise (*r* = –0.28, *P* = 0.01) and blue modules (*r* = –0.39, *P* < 0.05) were significantly related to LUSC and were thus selected. The turquoise module contained 797 genera, whereas the blue module contained 452 genera (Figure 1c and d).

Microbial co-regulatory networks were constructed using genera significantly related to LUSC in the blue and turquoise modules. The results showed that the microbial co-regulatory network of the blue module had 79 nodes (Figure 1e) and that of the turquoise module had 131 nodes (Figure 1f).

### 3.3 Genera related to prognosis

**Figure 2:**
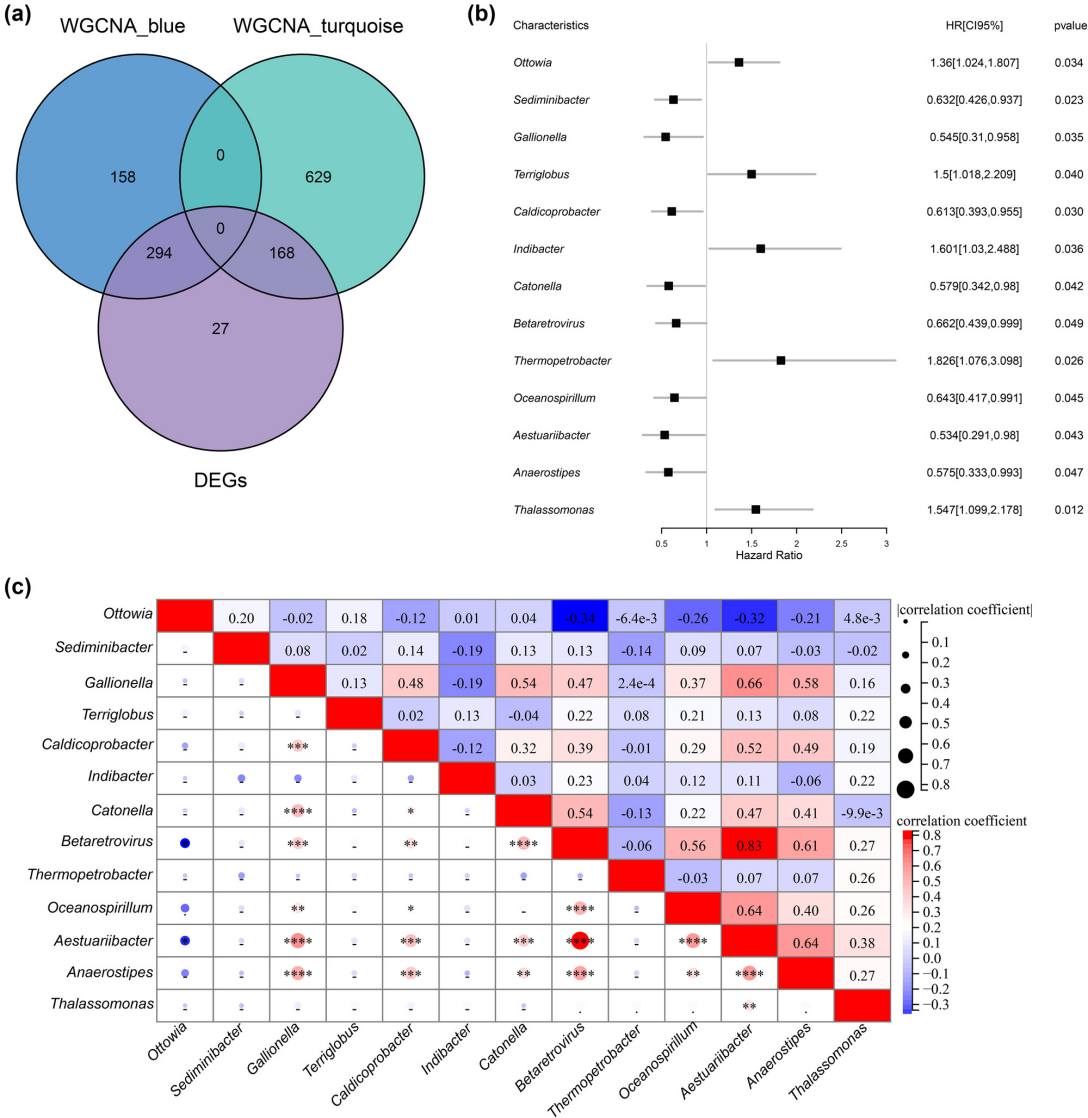
Analysis of prognosis-related microbial genera. (a) Venn diagram of the differentially abundant genera related to LUSC. (b) Univariate Cox regression analysis results revealing prognosis-related genera. (c) Correlation heat map of prognosis-related genera.

Venn analysis comparing the genera in the WGCNA modules and the differentially abundant genera showed that 462 genera were highly related to LUSC (Figure 2a). Univariate Cox regression analysis showed that 13 genera were significantly correlated with prognosis (*P* < 0.05) (Figure 2b). The Spearman correlation heat map of the 13 prognostic genera is shown in Figure 2c.

### 3.4 Construction of the MAPS model

After further screening of the 13 prognostic microbial genera using the stepwise multiple regression method, we obtained the optimal MAPS model. This included seven genera: *Sediminibacter*, *Terriglobus*, *Indibacter*, *Betaretrovirus*, *Thermopetrobacter*, *Oceanospirillum*, and *Thalassomonas*. The regression coefficients for the seven genera are listed in Table 1.

**Table 1:** Regression coefficient of characteristic bacteria in the optimal model

Genus	Coeff (fit.step)
*Sediminibacter*	−0.497
*Terriglobus*	0.625
*Indibacter*	0.781
*Betaretrovirus*	−0.611
*Thermopetrobacter*	0.476
*Oceanospirillum*	−0.486
*Thalassomonas*	0.437

**Figure 3:**
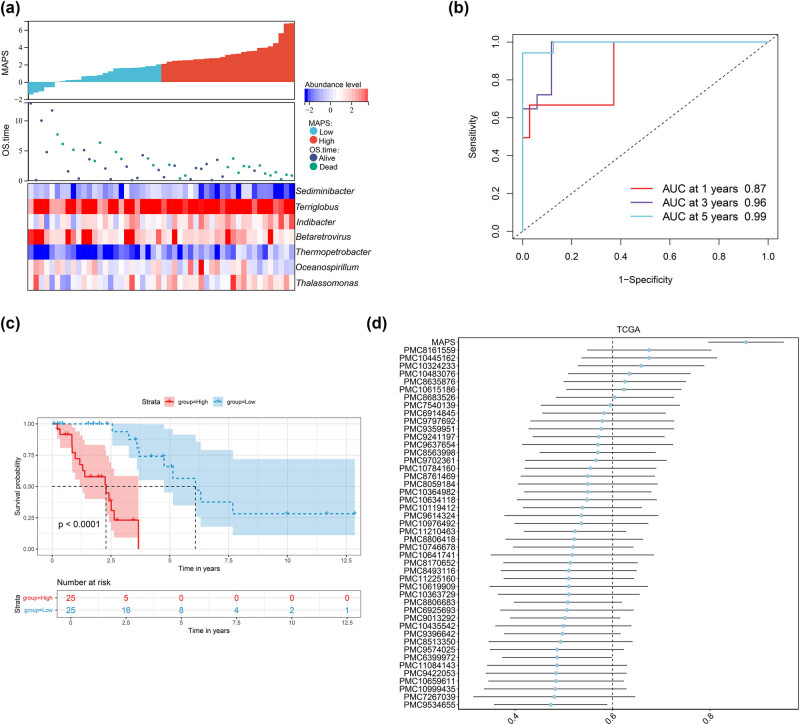
MAPS model analysis. (a) Sample distribution among the risk groups. (b) ROC curves showing the value of the MAPS model in predicting 1-, 3-, and 5-year survival times. (c) Kaplan–Meier curves revealing the overall difference between the two risk groups. (d) The C-indexes of the published signatures and MAPS model.

The samples were divided into high- and low-risk groups on the basis of the MAPS model. The sample distribution and abundance of model genera among the risk groups are shown in Figure 3a. The ROC curves revealed that the model had high 1-, 3-, and 5-year survival prediction values (AUC = 0.87, 0.96, and 0.99, respectively) (Figure 3b). The Kaplan–Meier curves showed that patients in the high-risk group had shorter survival times than those in the low-risk group (*P* < 0.0001; Figure 3c). Moreover, we compared the C-indexes of these published signatures with that of the MAPS model. The C-index of the MAPS model was the highest, suggesting that it has high predictive performance (Figure 3d).

### 3.5 Clinical characteristics of patients

**Figure 4:**
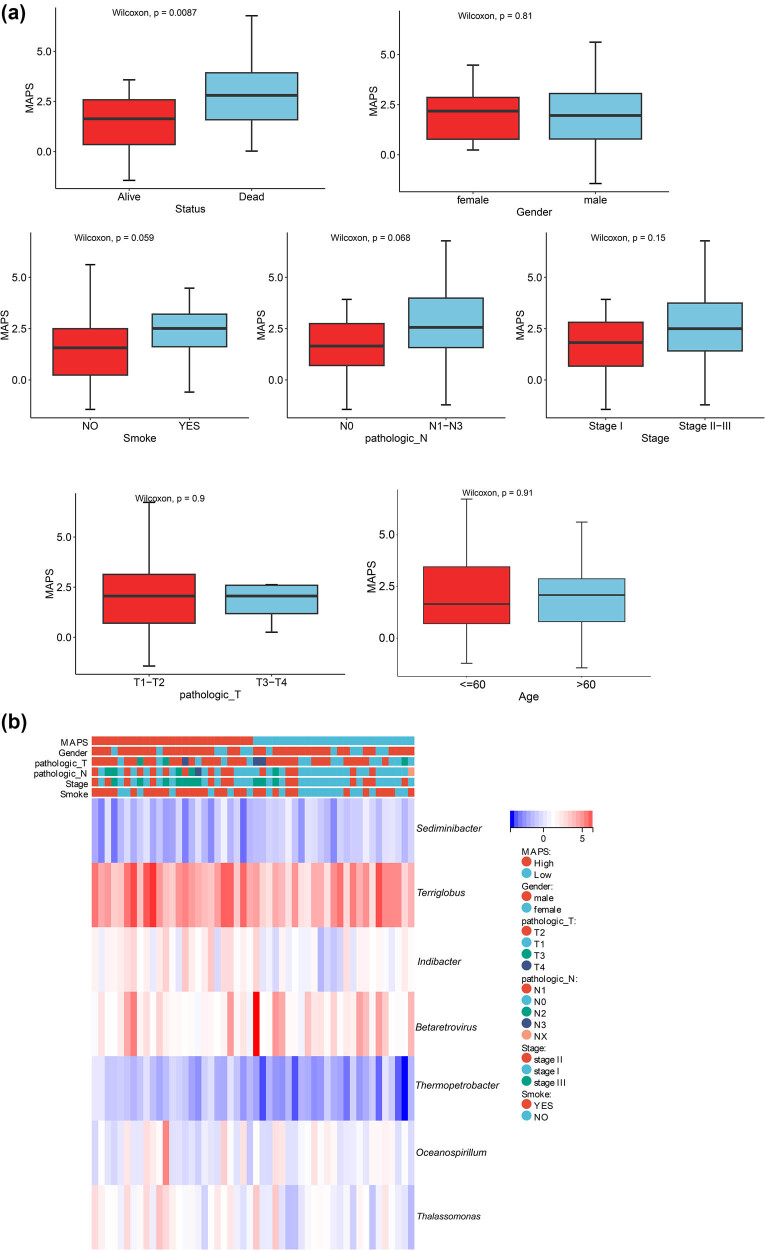
Association between the MAPS values and clinical features. (a) Difference in MAPS values between different clinical features (living status, sex, smoking, pathological_N, tumor stage, pathological_T, and age). (b) Heat map showing the correlations between the abundances of genera in the MAPS model and different clinical features.

**Figure 5:**
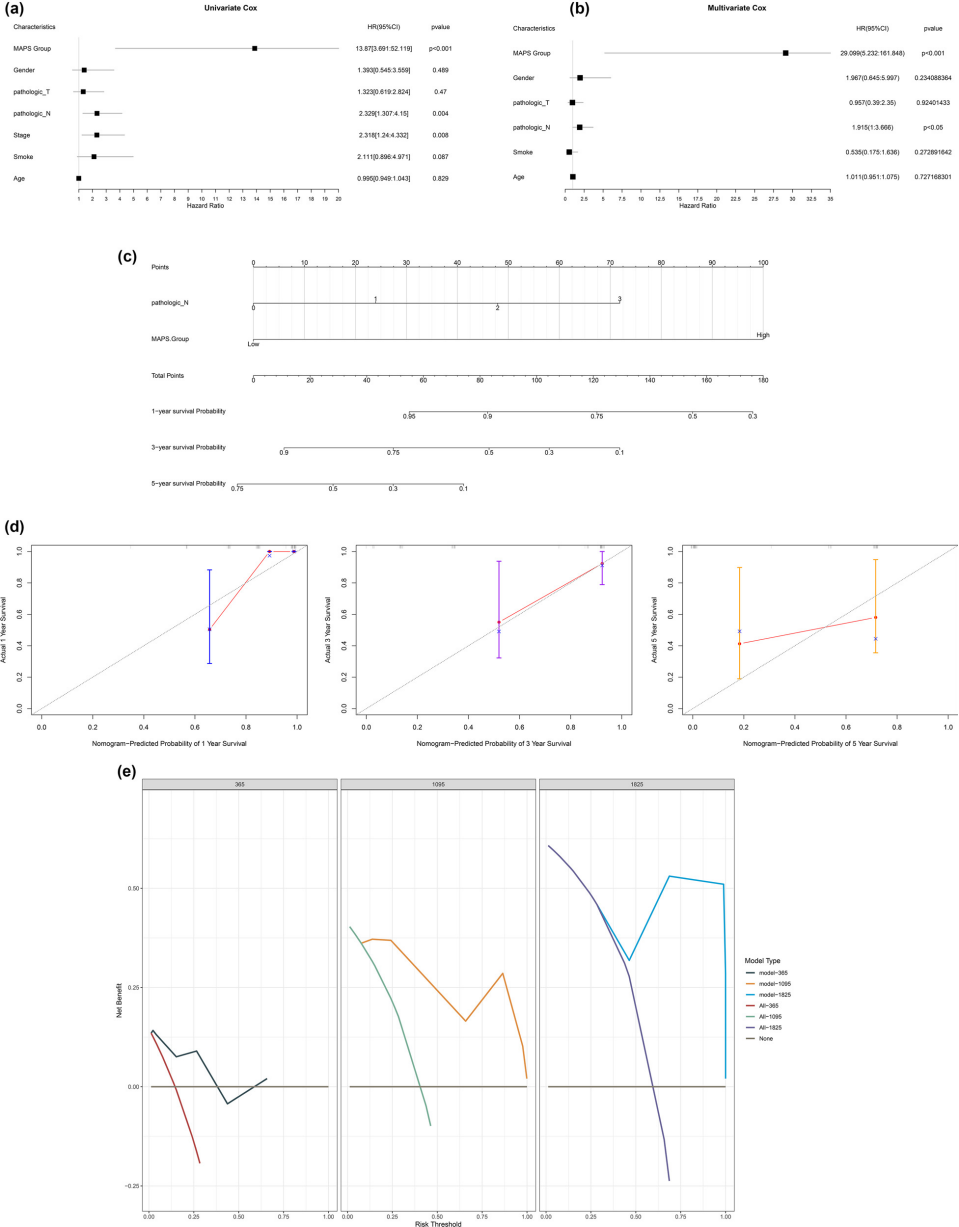
Prognosis analysis and nomogram construction. (a) Univariate Cox regression forest map. (b) Multivariate Cox regression forest map. (c) Nomogram constructed using independent prognostic factors. (d) Calibration curve. (e) Decision curve analysis results.

The risk scores of the MAPS model were analyzed using clinical data, including age, sex, pathological_T, pathological_N, tumor stage, smoking, and patient status. Significant differences in the MAPS scores were observed between the patient status (alive and dead) groups but not between the other groups of clinical factors (Figure 4a). The correlations between the abundances of genera in the MAPS model and different clinical features are shown in Figure 4b. Univariate Cox regression analysis revealed that pathological_N stage was a prognostic risk factor (Table 2, Figure 5a).

**Table 2:** Univariate Cox regression results

Characteristics	Hazard ratio	95% CI	*P* value
MAPS Group	13.87	(3.691:52.119)	*p* < 0.001
Gender	1.393	(0.545:3.559)	0.489
Pathologic_T	1.323	(0.619:2.824)	0.47
Pathologic_N	2.329	(1.307:4.15)	0.004
Stage	2.318	(1.24:4.332)	0.008
Smoke	2.111	(0.896:4.971)	0.087
Age	0.995	(0.949:1.043)	0.829

Considering that pathological_N and the tumor stage were confounding factors, sex, pathological_T, pathological_N, smoking, and age were included in a multivariate regression analysis for correction to ensure the reliability of the results (Table 3). According to the results, pathological_N and MAPS risk grouping were independent prognostic factors for patients with LUSC (Figure 5b). A nomogram was constructed using pathological_N and MAPS risk grouping, which showed high performance in predicting 1-, 3-, and 5-year survival (Figure 5c). The calibration curve and decision curve analyses confirmed the reliability of the nomogram (Figure 5d and e).

**Table 3:** Multivariate Cox regression results

Characteristics	Hazard ratio	95% CI	*P* value
MAPS Group	29.099	(5.232:161.848)	*p* < 0.001
Gender	1.967	(0.645:5.997)	0.234
Pathologic_T	0.957	(0.39:2.35)	0.924
Pathologic_N	1.915	(1:3.666)	*p* < 0.05
Smoke	0.535	(0.175:1.636)	0.273
Age	1.011	(0.951:1.075)	0.727

### 3.6 Abundance analysis of model genera

**Figure 6:**
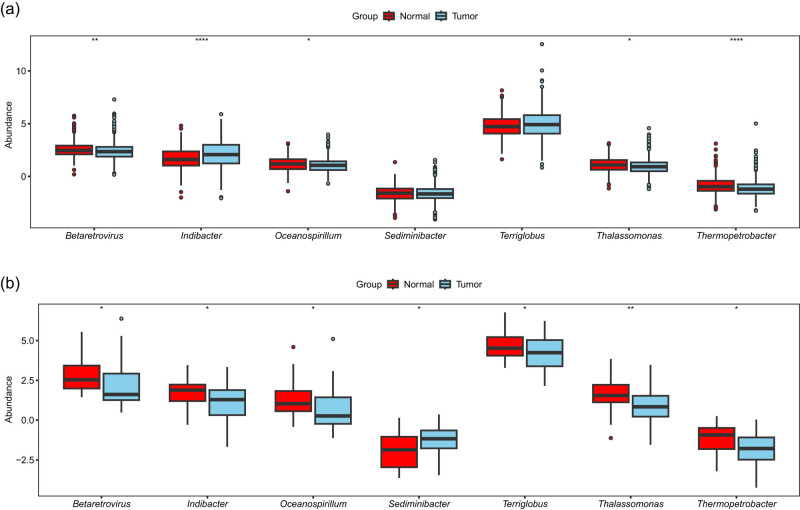
Differences in the abundance of model genera between tumor and normal tissue samples. (a) Box diagram showing the differences in abundance of model genera between LUAD and normal samples. (b) Box diagram showing the differences in abundance of model genera between LUSC and normal samples.

LUAD tumor microbiome data were downloaded from The Cancer Microbiome Atlas database, which includes 228 normal and 683 tumor tissues. Differences in the abundances of five genera (*Betaretrovirus*, *Indibacter*, *Oceanospirillum*, *Thalassomonas*, and *Thermopetrobacter*) were found between LUAD tumor tissues and normal tissues (Figure 6a). The abundance change trends of *Betaretrovirus*, *Oceanospirillum*, and *Thermopetrobacter* were consistent with those found in the LUSC samples (Figure 6b).

## 4 Discussion

In this study, we constructed a MAPS model using seven intratumoral microbial genera: *Sediminibacter*, *Terriglobus*, *Indibacter*, *Betaretrovirus*, *Thermopetrobacter*, *Oceanospirillum*, and *Thalassomonas*. This model showed high performance in predicting the prognosis of LUSC. *Indibacter*, *Oceanospirillum*, *Thalassomonas*, and *Thermopetrobacter* were the key genera in both LUSC and LUAD.

As one of the common malignant tumors threatening human health, lung cancer has a high incidence, a complicated pathogenesis, no obvious symptoms in the early stages, and poor treatment effects [22]. It is the main cause of cancer morbidity and mortality in men and ranks third in terms of incidence and second in terms of mortality among women [23]. Although minimally invasive surgery, chemotherapy, and targeted therapy for this disease have progressed in recent years, the 5-year survival rate of patients with lung cancer remains unsatisfactory, being only between 10 and 20% in most areas worldwide [24]. If benign and malignant lung shadows can be distinguished earlier and more accurately, patients can be operated on early to improve their 5-year survival rate.

The rapid development of next-generation sequencing approaches has aided the revelation that even healthy lungs have unique and complex microbial communities [25,26]. *Pseudomonas*, *Streptococcus*, *Clostridium*, *Megacoccus*, and *Clostridium* cocci are dominant in healthy lungs [27,28]. Various microorganisms colonize the lungs and are closely associated with the development of respiratory diseases. Moreover, microorganisms in the lungs are clearly associated with high risk factors for lung cancer. Studies have shown that the distribution of lung microbiotas may differ among patients with NSCLC with different clinicopathological characteristics [29] and that the lung microflora may affect the development of NSCLC [30]. The microbial characteristics of local tumors are biomarkers for predicting the response of NSCLC cells to immune checkpoint inhibitors and evaluating the disease prognosis [31]. In two recent studies, bronchoalveolar lavage fluids were sampled from patients with lung cancer, patients with benign lung lesions, and healthy individuals for metagenomic sequencing [32,33]. *Bacteroides*, *Proteus*, *Actinomycete*s, and *Firmicutes* were detected in all the samples. However, in the patients with lung cancer, the abundances of these microbes were significantly decreased and *Proteobacteria* was the dominant genus, which was significantly different from the abundances of genera in the other two groups [32,33]. Hosgood et al. [9] sequenced the 16S rRNA gene in the sputum of nonsmoking female patients with lung cancer in Xuanwei, China (an area with coal-burning exposure). They found that the increased risk of lung cancer was related to a lower α diversity, and a decreased microbial diversity was related to the risk of lung cancer. Other studies have demonstrated significant differences in microbial species and their abundances between benign and malignant lung lesions [34,35].

Currently, the mechanisms underlying the pathogenic role of microbial communities in the lungs are not well understood. However, some researchers speculate that the microecological balance in the lung is broken under pathological conditions; that is, a “steady imbalance” exists, which may be determined by the following three factors [36]: (a) the abnormal lung function leads to a deficiency in the immune defense of the host; (b) the abnormal internal environment of the lung promotes the disease growth, which leads to changes in the microbial community structure in the lungs; and (c) some microorganisms can recognize host signaling molecules, including hormones, neurotransmitters, and cytokines, and the changes in these molecules can affect the microbial community structure. Therefore, the lung microbiota can reflect the health status of the organ, activate and aggregate immune cells with immunomodulatory activity, and participate in the formation of an immune defense environment [37,38]. Huang et al. [29] performed 16S rRNA gene sequencing on bronchial lavage fluid and sputum samples from patients with different stages of LUSC and LUAD. The results showed that the abundances of *Actinobacillus* and *Arthrobacter* were higher in patients with early LUAD than in individuals with early LUSC. In our study, we found that the tumor stage was a prognostic risk factor, similar to the results of other studies.

This study had some limitations. The heterogeneity of lung cancer and the objective factors existing in clinical research (including race, age, sex, smoking status, antibiotic use, and other respiratory diseases) require large-scale sample-size studies to ensure sufficient statistical tests to explain the correlation between lung microorganisms and lung cancer. Additionally, the use of healthy lung tissue as a baseline for microbial comparison may not be optimal. Some bacterial colonization in healthy lung tissue could be associated with opportunistic microorganisms that arise during episodes of lung inflammation or other respiratory conditions. This could introduce confounding factors and make it difficult to distinguish between the microbial composition associated with true lung health and that influenced by inflammation or transient microbial colonization. Moreover, crucial data on tissue necrosis and inflammation, which could significantly influence the microbiome, were not available in the datasets used. Accurate and specific detection methods should be used in future studies on lung microorganisms to identify differential proteins. Furthermore, more accurate methods need to be developed for the diagnosis and treatment of respiratory diseases, such as lung cancer. Obtaining the characteristics of the lung microbiome in patients with lung cancer is of great significance for improving early screening of the disease, curative effect monitoring, follow-up observation, and individualized medication.

## 5 Conclusion

Our results indicated that the MAPS model may be a powerful prognostic biomarker for LUSC. Moreover, *Indibacter*, *Oceanospirillum*, *Thalassomonas*, and *Thermopetrobacter* may be the key intratumoral microorganisms involved in the development of LUSC and LUAD.

## Supplementary Material

Supplementary Table
